# Development and external validation of a prediction risk model for short-term mortality among hospitalized U.S. COVID-19 patients: A proposal for the COVID-AID risk tool

**DOI:** 10.1371/journal.pone.0239536

**Published:** 2020-09-30

**Authors:** Kaveh Hajifathalian, Reem Z. Sharaiha, Sonal Kumar, Tibor Krisko, Daniel Skaf, Bryan Ang, Walker D. Redd, Joyce C. Zhou, Kelly E. Hathorn, Thomas R. McCarty, Ahmad Najdat Bazarbashi, Cheikh Njie, Danny Wong, Lin Shen, Evan Sholle, David E. Cohen, Robert S. Brown, Walter W. Chan, Brett E. Fortune

**Affiliations:** 1 Division of Gastroenterology and Hepatology, Weill Cornell Medicine, New York-Presbyterian Hospital, New York, NY, United States of America; 2 Joan & Sanford I. Weill Medical College, Weill Cornell Medicine, New York, NY, United States of America; 3 Department of Medicine, Brigham and Women’s Hospital, Boston, MA, United States of America; 4 Harvard Medical School, Boston, MA, United States of America; 5 Division of Gastroenterology, Hepatology and Endoscopy, Brigham and Women’s Hospital, Boston, MA, United States of America; 6 Department of Research Informatics, Information Technologies Services, Weill Cornell Medicine, New York-Presbyterian Hospital, New York, NY, United States of America; National Yang-Ming University, TAIWAN

## Abstract

**Background:**

The 2019 novel coronavirus disease (COVID-19) has created unprecedented medical challenges. There remains a need for validated risk prediction models to assess short-term mortality risk among hospitalized patients with COVID-19. The objective of this study was to develop and validate a 7-day and 14-day mortality risk prediction model for patients hospitalized with COVID-19.

**Methods:**

We performed a multicenter retrospective cohort study with a separate multicenter cohort for external validation using two hospitals in New York, NY, and 9 hospitals in Massachusetts, respectively. A total of 664 patients in NY and 265 patients with COVID-19 in Massachusetts, hospitalized from March to April 2020.

**Results:**

We developed a risk model consisting of patient age, hypoxia severity, mean arterial pressure and presence of kidney dysfunction at hospital presentation. Multivariable regression model was based on risk factors selected from univariable and Chi-squared automatic interaction detection analyses. Validation was by receiver operating characteristic curve (discrimination) and Hosmer-Lemeshow goodness of fit (GOF) test (calibration). In internal cross-validation, prediction of 7-day mortality had an AUC of 0.86 (95%CI 0.74–0.98; GOF p = 0.744); while 14-day had an AUC of 0.83 (95%CI 0.69–0.97; GOF p = 0.588). External validation was achieved using 265 patients from an outside cohort and confirmed 7- and 14-day mortality prediction performance with an AUC of 0.85 (95%CI 0.78–0.92; GOF p = 0.340) and 0.83 (95%CI 0.76–0.89; GOF p = 0.471) respectively, along with excellent calibration. Retrospective data collection, short follow-up time, and development in COVID-19 epicenter may limit model generalizability.

**Conclusions:**

The COVID-AID risk tool is a well-calibrated model that demonstrates accuracy in the prediction of both 7-day and 14-day mortality risk among patients hospitalized with COVID-19. This prediction score could assist with resource utilization, patient and caregiver education, and provide a risk stratification instrument for future research trials.

## Introduction

The 2019 novel coronavirus disease (COVID-19), caused by severe acute respiratory syndrome coronavirus 2 (SARS-CoV-2) has become an international pandemic. Although the original outbreak was attributed to zoonotic transmission in Wuhan, China, human-to-human transmission through respiratory droplets and aerosolization has resulted in rapid disease spread across the world. As of May 11, 2020 there have been over 4 million confirmed cases of COVID-19, with many more likely infected, and more than 280,000 associated deaths worldwide [1]. Clinical presentations of COVID-19 have been heterogeneous, ranging from mild flu-like symptoms (fever, cough, and fatigue) to severe respiratory symptoms and hypoxia resulting in acute respiratory distress syndrome (ARDS). Given the wide spectrum of symptoms, there have been varied clinical trajectories, ranging from outpatient management to hospital admission, need for intensive care and/or mechanical ventilation, multisystem organ failure, and death. In select cases, the progression of disease may be extremely rapid, with the observed time between onset of symptoms and the development of ARDS as short as 9 days [2].

With a vast number of individuals affected by the disease, there has been an imbalance between the supply and demand of hospital and intensive care unit (ICU) beds, straining available healthcare resources [3]. Attempts have been made to clarify the relationship of risk factors with clinical prognosis in order to risk stratify patients and appropriately allocate available but limited healthcare resources. Several observational studies have noted that patients who are older or carry various comorbidities, such as diabetes, cardiovascular disease [4], and hypertension [5, 6], have higher risk for in-hospital mortality from COVID-19 [7]. Other studies have shown certain biomarkers such as ferritin, lactate dehydrogenase (LDH), D-dimer, and C-reactive protein (CRP) to predict COVID-19 severity [8, 9]. While there have been attempts at creating prediction models that combine several variables to estimate prognosis, including the use of scoring systems and machine learning [10], many of these models have been suboptimal due to high-risk of bias, restricted sample sizes, and limited number of outcomes of interest [10].

Given the paucity of comprehensive data to guide providers and caregivers on the prognosis of COVID-19 patients and the potential for rapid disease progression, there remains a need to develop a prediction model for mortality. As New York City has become the epicenter of the COVID-19 pandemic in the United States, we sought to use our large cohort of patients to develop a prognostic model that could predict the risk of death within 7 or 14 days from admission. Using data from hospitalized patients infected with COVID-19 from two New York City hospitals, the primary objective of this study is to construct an accurate prognostic model, called the COVID-AID (Admission to Death) risk tool, and externally validate the model using another large cohort from a different region of the United States.

## Material and methods

### Patient population and data collection for independent variables

This was a retrospective study performed at two hospitals in Manhattan (an academic tertiary referral center and a smaller community hospital). Adult patients (age ≥18 years) with a positive real-time reverse-transcription polymerase chain reaction (RT-PCR) from a respiratory sample (naso- or oropharyngeal, or bronchial/sputum samples) for SARS-CoV-2 between March 4, and April 9, 2020 were included. All included patients had laboratory-confirmed COVID-19. Patients who were admitted (including temporary observation defined as admission to emergency department and discharge within 24 hours) were included in this analysis. The study was reviewed and approved by the institutional review board (Weill Cornell Medicine: 2004021793). We followed TRIPOD guidelines for reporting multivariable prediction models (See S1 Table in S1 File) [11].

Clinical parameters including demographics (age, gender, race/ethnicity) and past medical history including cancer, chronic kidney disease (CKD), chronic obstructive pulmonary disease (COPD), asthma, obstructive sleep apnea (OSA), cardiovascular disease (CVD) [4], history of venous thromboembolism (VTE), diabetes, hypertension, inflammatory bowel disease (IBD), chronic liver disease, or solid organ transplantation were obtained from patients’ medical records.

Date of first symptoms recorded and date of positive SARS-CoV-2 PCR were recorded, as were the initial vital signs upon presentation. The first set of recorded vital signs including temperature (with fever defined as T ≥37.8), respiratory rate (RR), heart rate (HR), systolic, diastolic, and mean arterial pressures (SBP, DBP, MAP, respectively), and body mass index (BMI) were extracted. BMI was categorized into normal weight between a BMI ≥18.5 and <25 kg/m^2^ (reference category), underweight BMI <18.5 kg/m^2^, overweight BMI ≥25 kg/m^2^ and <30 kg/m^2^, obese BMI ≥30 kg/m^2^ and <40 kg/m^2^, and morbidly obese BMI ≥40 kg/m^2^.

A comprehensive set of laboratory studies was also extracted upon admission. This included complete blood count (white blood cell, absolute neutrophil, absolute lymphocyte, and platelet counts), serum creatinine (sCr), liver tests including alanine aminotransferase (ALT), aspartate aminotransferase (AST), total bilirubin, alkaline phosphatase, and albumin, as well as serum troponin, procalcitonin, lactate dehydrogenase, fibrinogen, lactate levels, and inflammatory markers including C reactive protein (CRP), D-dimer, and ferritin. Patients were considered to have biochemical indication of liver injury at presentation if they had ALT or AST>40 U/L, total bilirubin>1.2 mg/dL, or alkaline phosphatase>150 U/L (upper limit of normal at our laboratory). Kidney dysfunction was defined as Kidney Disease Improving Global Outcomes Acute Kidney Injury (KDIGO AKI) stage 2 or greater where sCr was at least 2 times or more then reference value with reference estimate at 1mg/dL (i.e. sCr ≥ 2.0 mg/dL) [12].

Patients’ degree of hypoxia on admission was categorized based on pulse oximetry as a) no hypoxia (defined as an oxygen saturation of ≥95% on room air), b) moderate hypoxia (defined as maintaining an oxygen saturation of 90–95% on room air or ≥90% with 4 liters or less supplemental oxygen through a nasal cannula, or c) severe hypoxia (defined as needing more than 4 liters of supplemental oxygen, non-rebreather mask or non-invasive (e.g. BiPAP) or invasive ventilation to maintain an oxygen saturation of ≥90%, or failure to maintain an oxygen saturation of ≥90%).

#### Outcomes

Data were extracted regarding need for supplementary oxygen, non-invasive positive pressure ventilation (NIPPV), or invasive ventilatory support with mechanical ventilation, ICU admission, and death. The main outcome of this study was 14-day mortality. The secondary outcome of interest was 7-day mortality.

#### Follow-up, survival modeling, and prediction

Patients were followed from time of admission until May 24th, 2020. Survival time was defined as the time between admission to death (failure time) or the date when patients were last known to be alive (censoring time). To calculate 7-day mortality, deaths that occurred within 7 days of admission were kept while deaths occurring after 7 days were recorded as non-events. To calculate 14-day mortality, deaths that occurred within 14 days of admission were kept while deaths occurring after 14 days were recorded as non-events.

Univariable logistic regression models were created for each of the aforementioned independent variables. To lessen the influence of extreme values, we transformed continuous variables into natural logarithms. Selection of predictors was performed using Chi-square automatic interaction detection (CHAID) modeling in order to decrease the dimensionality of the data and explore the most informative variables for identifying patient groups with the highest risk of mortality [13]. Variables which were significant in univariable analysis (defined as p-value <0.05) were included in the CHAID model with adjusted significance testing (Bonferroni method) without limitation on the number of nodes and branches [14, 15]. Independent risk factors that were chosen from the CHAID algorithm (X_j_) were subsequently included in a multivariable logistic regression and their regression coefficients (β_j_) were stored. An individual odds ratio of mortality (OR_i_) was calculated for each patient by adding the product of each individual risk factor level and its corresponding coefficient:

$$OR_{i=}exp\;(\sum_{1\;to\;j}[X_{ij}\;\beta_{j}]+\beta_{0})$$

Where OR_i_ is each individual's OR of mortality, X_ij_ is the individual's level of j'th risk factor, and β_j_ is the coefficient for the j'th risk factor, and β_0_ is the intercept for the logistic regression. An individual probability of death (P_i_) was calculated from the individual’s OR_i_:

$$P_{i}=OR_{i}/(1+OR_{i})$$

### Internal and external validation of the prediction model

10-fold cross validation was used for internal validation of the prediction models, and mean (95%CI) performance characteristics across 10 internal validations as well as overall performance characteristics in the whole development cohort were reported [16]. Discriminant analyses in the internal and external validation sets were performed using receiver operating characteristic (ROC) curve. Area under the curve (AUC) and its 95% confidence intervals were reported. Calibration was performed using visual calibration plots of observed versus predicted risk of death within groups formed by 10 quantiles of predicted risk of death, with overlying linear predictions and their 95% confidence intervals. Chi-Squared statistics and corresponding p-values (DF = 8) were reported from the Hosmer-Lemeshow goodness-of-fit test for calibration.

External validation was separately performed by using a cohort of 265 adult patients (age> = 18) admitted with a positive RT-PCR for SARS-CoV-2 from a respiratory sample between March 7, and April 2, 2020, in 2 tertiary care and 7 community hospitals from a single healthcare system in Massachusetts. Identical definitions, independent, and outcome variables were used in the external validation analysis.

The study was reviewed and approved by the corresponding institutional review board (Partners Healthcare: 2020P0000983).

Chained multiple imputations (50 repetitions) using linear and logistic regressions for continuous and categorical variables, respectively, were used to impute missing data on independent variables (S2 Table in S1 File) [17]. All tests were two-tailed with a significance level of alpha < 0.05, except when adjusted for multiple comparisons as described above. All analyses were performed with Stata 13.0 for Windows (StataCorp LP, College Station, TX). The 3D graphs of risk were generated using Microsoft Mathematics (Microsoft Corporation, Redmond, WA).

## Results

**Fig 1:**
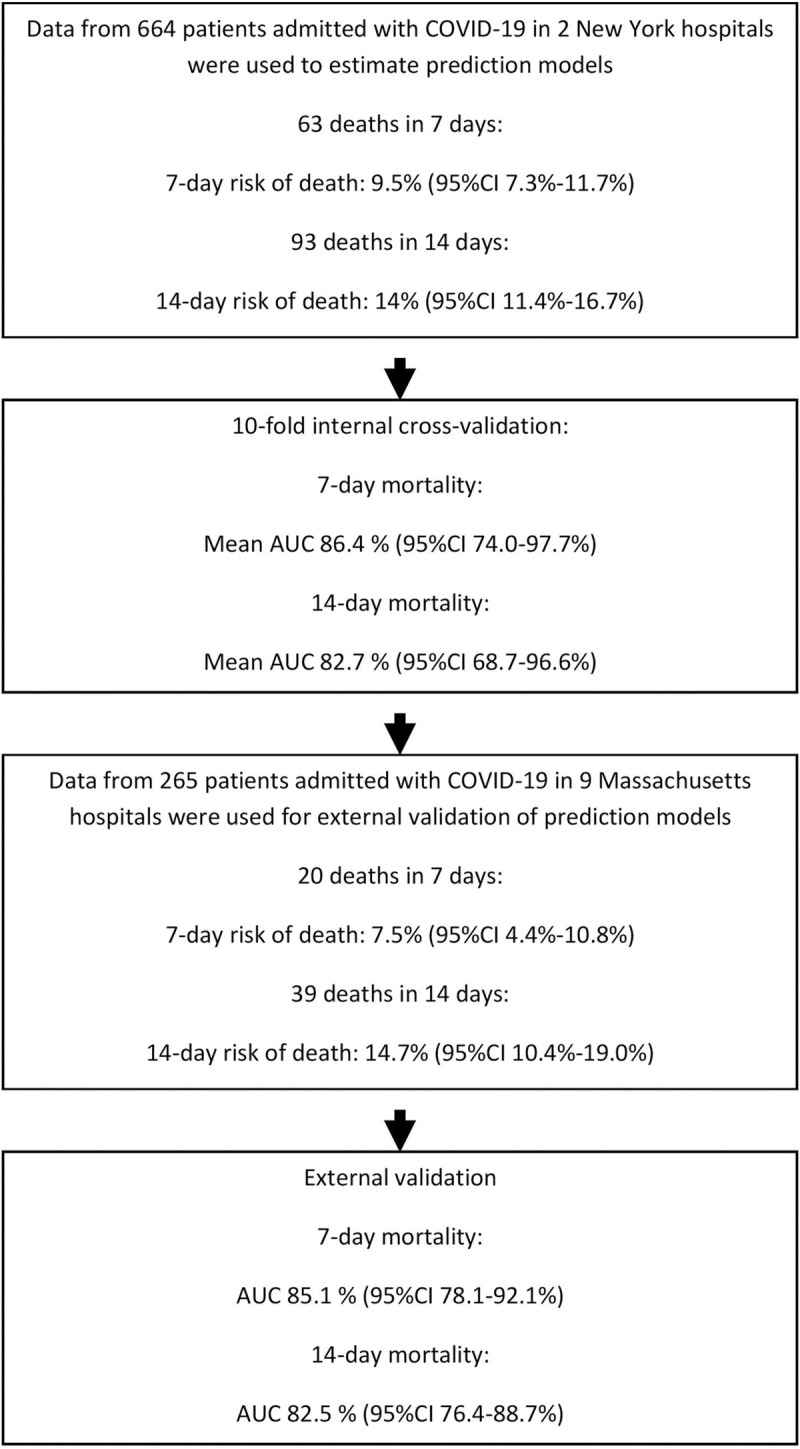
Flowchart of the populations used to build and externally validate mortality prediction models.

A total of 664 patients were hospitalized with COVID-19 between March 4, and April 9, 2020. The mean age of patients was 64 years (SD = 17), with 63% being male (Table 1). Ninety-three deaths occurred within 14 days of hospital admission and observed 7-day and 14-day mortality rates were 9.5% (95%CI 7.3–11.7%), and 14% (95%CI 11.4–16.7%), respectively (Fig 1).

**Table 1:** Demographics, medical history, laboratory, and clinical findings of patients with COVID-19 upon admission.

Variable	Total, n = 664	Death within 14 days of admission		P-value*
		No, n = 571	Yes, n = 93	
**Age, years**	**64±17**	**62±16**	**80±12**	**<0.001**
Male	415(63)	357(63)	58(62)	0.977
**Race/ethnicity**				**0.008**
**White/Caucasian**	**217(44)**	**183(43)**	**34(50)**	
**Black/African-American**	**73(15)**	**64(15)**	**9(13)**	
**Asian**	**68(14)**	**52(12)**	**16(24)**	
**Other**	**134(27)**	**125(29)**	**9(13)**	
BMI				
Normal	148(31)	120(29)	28(43)	0.084
Underweight	18(4)	14(3)	4(6)	
Overweight	142(30)	124(30)	18(28)	
Obese	139(29)	127(31)	12(18)	
Morbidly obese	32(7)	29(7)	3(5)	
Pre-existing comorbidities				
**Hypertension**	**377(57)**	**309(54)**	**68(73)**	**0.001**
Diabetes	206(31)	174(30)	32(34)	0.447
**Chronic kidney disease**	**92(14)**	**69(12)**	**23(25)**	**0.001**
**Cardio-vascular disease**	**135(20)**	**98(17)**	**37(40)**	**<0.001**
COPD/Asthma	81(12)	69(12)	12(13)	0.823
Obstructive sleep apnea	27(4)	23(4)	4(4)	0.902
VTE	54(8)	43(8)	11(12)	0.163
Cancer	78(12)	62(11)	16(17)	0.081
IBD	8(1)	7(1)	1(1)	0.902
Chronic liver disease	20(3)	18(3)	2(2)	0.602
Solid organ transplantation	20(3)	18(3)	2(2)	0.602
Vital signs				
fever	170(26)	153(27)	17(18)	0.082
**Respiratory rate**	**21±6**	**21±5**	**24±7**	**<0.001**
Heart rate	94±19	94±19	92±19	0.450
**Mean arterial pressure, mmHg**	**93±14**	**94±14**	**89±15**	**0.001**
**Hypoxia on presentation**				**<0.001**
**No**	**248(37)**	**228(40)**	**20(22)**	
**Moderate**	**223(34)**	**196(34)**	**27(29)**	
**severe**	**193(29)**	**147(26)**	**46(49)**	
Kidney dysfunction**	**93(14)**	**62(11)**	**31(33)**	**<0.001**
Laboratory findings				
**Creatinine, mg/dL**	**1.6±3.1**	**1.5±3.1**	**2.3±2.3**	**<0.001**
White blood cell count, x10^3	7.7±6.6	7.6±6.8	8.0±4.6	0.673
Absolute lymphocyte count,x10^3	1.1±2.1	1.1±2.2	0.8±0.6	0.053
Absolute neutrophil count, x10^3	7±9.2	6.7±9.0	8.3±10.6	0.056
**Platelet count, x10^3**	**218±99**	**223±100**	**188±92**	**0.001**
**Procalcitonin, ng/mL**	**0.9±4.3**	**0.7±3.1**	**1.9±8.4**	**<0.001**
**D-dimer, ng/mL**	**1736±4600**	**1495±4321**	**3222±5874**	**<0.001**
**C reactive protein, mg/dL**	**15±13**	**15±14**	**17±10**	**0.015**
**Lactate dehydrogenase, U/L**	**475±302**	**450±220**	**633±578**	**<0.001**
**Lactate, mmol/L**	**1.9±1.5**	**1.7±1.1**	**2.7±2.6**	**<0.001**
**Ferritin, ng/mL**	**1273±1780**	**1107±1215**	**2265±3501**	**0.001**
**Troponin I, ng/mL**	**0.2±0.9**	**0.1±0.8**	**0.5±1.4**	**<0.001**
Albumin, g/dL	3.3±0.6	3.3±0.7	3.2±0.6	0.225
Total bilirubin, mg/dL	0.7±0.5	0.7±0.5	0.7±0.5	0.516
ALT, U/L	49±53	49±51	46±61	0.021
**AST, U/L**	**61±76**	**56±54**	**90±151**	**<0.001**
Alkaline phosphatase, U/L	90±78	90±83	85±46	0.461

^Data are mean ± SD, or n(%).

*P-values are from a univariable logistic regression model with 14-day mortality as the outcome. The continuous variables are transformed by natural logarithm before used in regression. VTE: venous thromboembolism; IBD: inflammatory bowel disease; NSAID: non-steroidal anti-inflammatory drug; AST: aspartate aminotransferase; ALT: alanine aminotransferase; INR: international normalized ratio; aPTT: activated partial thromboplastin time.

**Kidney dysfunction, defined as serum creatinine at admission ≥ 2 mg/dL

### Analysis of mortality risk factors

In univariable analysis, age, race/ethnicity, history of hypertension or cardiovascular disease, history of chronic kidney disease, mean arterial pressure (MAP), respiratory rate and presence of hypoxia on presentation, serum creatinine level, presence of kidney dysfunction, platelet count, procalcitonin, lactate dehydrogenase, lactic acid, troponin, Ferritin, D-dimer, C-reactive protein, and AST levels on presentation were significantly associated with 14-day mortality (Table 1).

**Fig 2:**
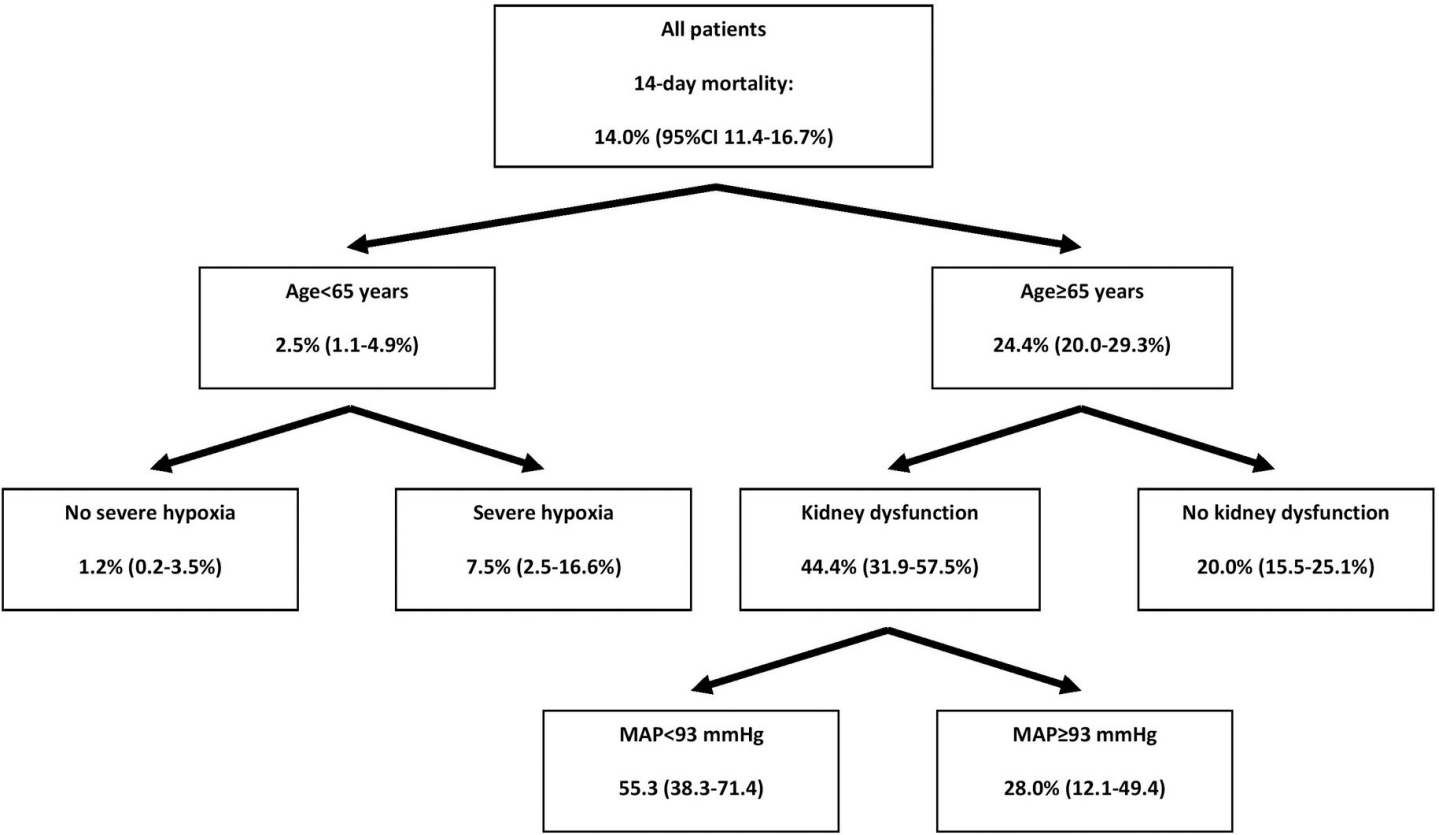
Optimal decision tree for categorizing patients admitted for COVID-19 based on the most informative predictors of 14-day mortality.

These variables were then included in the CHAID algorithm to find an optimal decision tree for splitting patients into low- and high-risk categories and predicting the risk of death. Age, admission MAP, presence of severe hypoxia (compared to no or moderate hypoxia) on presentation, and presence of kidney dysfunction on admission were selected as the most informative risk factors for categorizing patients according to their risk of 14-day mortality (Fig 2).

### Development of 7-day and 14-day mortality prediction models

These four risk factors (age, MAP, presence of severe hypoxia, presence of kidney dysfunction) were then included in two separate multivariable regression models, one with 7-day mortality as the outcome, and another with 14-day mortality as the outcome, in order to estimate the corresponding coefficient for each risk factor and outcome pair. The details of the regression parameters are included in Table 2.

**Table 2:** Risk prediction model for mortality risk for patients admitted with COVID-19, developed using the training data set.

7-day mortality			14-day mortality		
Risk factor	Coefficient	Standard error	Risk factor	Coefficient	Standard error
Log_e_(age)	7.8913	1.2109	Log_e_(age)	6.4001	0.8852
Log_e_(MAP)	-1.5434	0.9283	Log_e_(MAP)	-1.2073	0.7999
Kidney dysfunction*	1.0738	0.3445	Kidney dysfunction	1.0281	0.3038
Severe hypoxia	0.9385	0.3087	Severe hypoxia	0.7977	0.26
Intercept	-29.8233	7.1248	Intercept	-24.17	5.5901

*Kidney dysfunction, defined as serum creatinine at admission ≥ 2 mg/dL

Each patient’s predicted odds of 7-day mortality was then calculated as

OR_7-day_ = exp [Log_e_(age)* 7.8913 + Log_e_(MAP)* -1.5434 + 1.0738 (if kidney dysfunction present) + 0.9385 (if severe hypoxia present) -29.8233]

Similarly, each patient’s predicted odds of 14-day mortality was calculated as

OR_14-day_ = exp [Log_e_(age)* 6.4001+ Log_e_(MAP)* -1.2073+ 1.0281 (if kidney dysfunction present)+ 0.7977 (if severe hypoxia present) -24.1700]

The predicted odds were then translated into predicted probabilities for each patient (S3 Table in S1 File and S1 and S2 Figs).

### Internal validation

**Fig 3:**
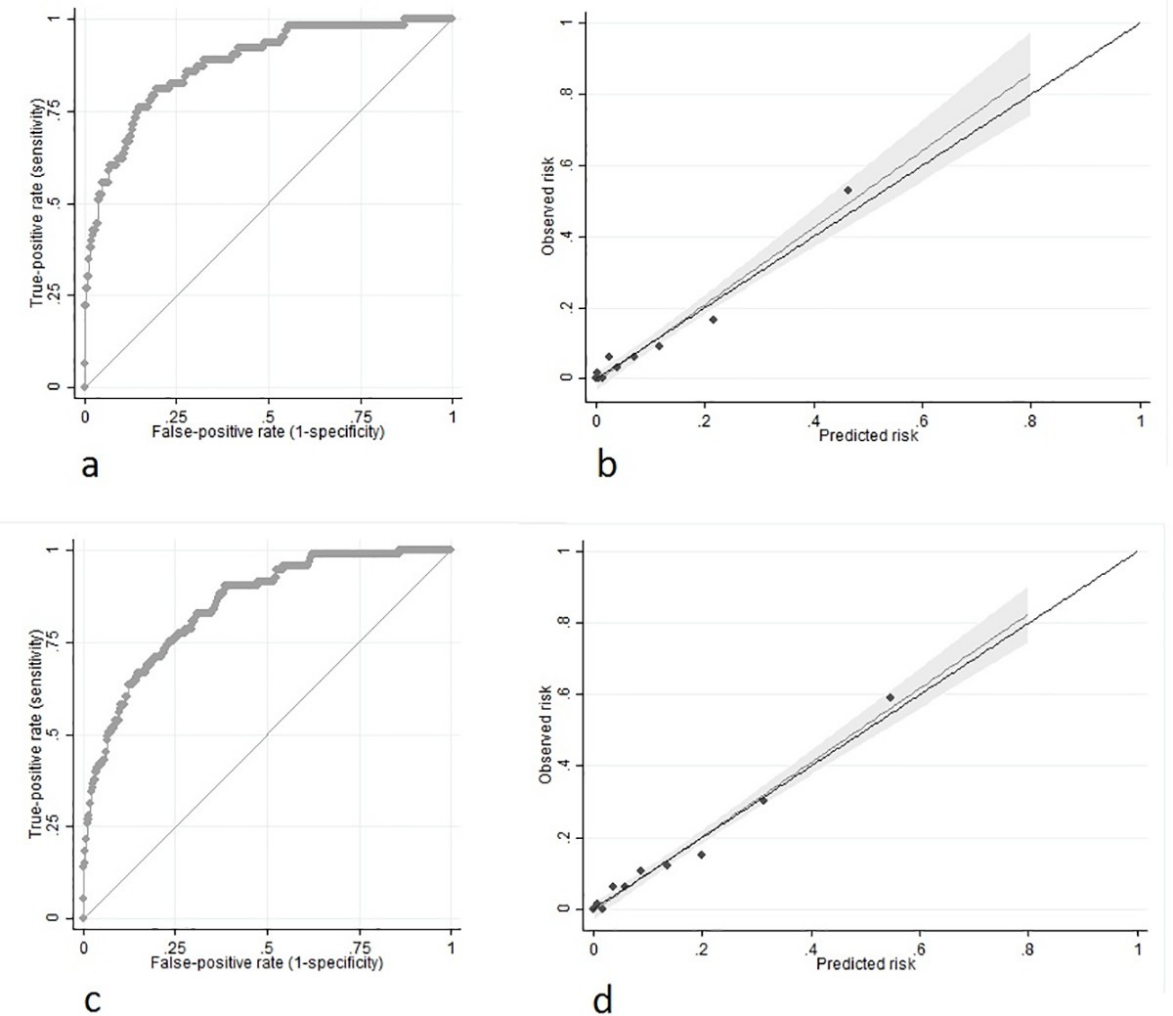
Discrimination and calibration of prediction models in internal validation. a) 7-day mortality: Receiver operator characteristic (ROC) curve for discrimination. Area under the curve (AUC) = 0.877 (95%CI 0.831–0.923). b) 7-day mortality: Calibration plot of observed versus predicted risk of mortality (Hosmer-Lemeshow chi-squared = 9.10, p = 0.334; DF = 8). c) 14-day mortality: Receiver operator characteristic (ROC) curve for discrimination. Area under the curve (AUC) = 0.847 (95%CI 0.806–0.888). d) 14-day mortality: Calibration plot of observed versus predicted risk of mortality (Hosmer-Lemeshow chi-squared = 9.64, p = 0.291; DF = 8).

10-fold internal cross validation showed a mean AUC of 0.864 (95%CI 74.0–97.7%) and a mean Hosmer-Lemeshow chi-squared statistic of 5.13 (p = 0.744) for prediction of 7-day mortality. The mean AUC was 0.827 (95%CI 0.687–0.966) with a Hosmer-Lemeshow chi-squared statistic of 6.53 (p = 0.588) for prediction of 14-day mortality across 10 internal cross validations. The model had excellent overall discrimination for 7-day mortality with an AUC of 0.877 (95%CI 0.831–0.923; Fig 3A) in the development cohort. The model also had excellent discrimination for 14-day mortality in the development cohort with an AUC of 0.847 (95%CI 0.806–0.888; Fig 3C). The models showed excellent calibration for predicting both 7-day (Hosmer-Lemeshow chi-squared = 9.10, p = 0.334; DF = 8), and 14-day (Hosmer-Lemeshow chi-squared = 9.64, p = 0.291; DF = 8) mortality, with excellent agreement between observed and predicted risk of mortality across 10 quintiles of risk (Fig 3B and 3D).

### External validation

The external validation cohort consisted of 265 patients admitted with laboratory-confirmed COVID-19. Patients in this cohort had a mean age of 65 years (SD = 17), and were 56% male. 39 deaths occurred within 14 days of admission and 7-day and 14-day mortality rates were 7.5% (95%CI 4.4–10.8%) and 14.7% (95%CI 10.4–19.0%), respectively (Fig 1).

**Fig 4:**
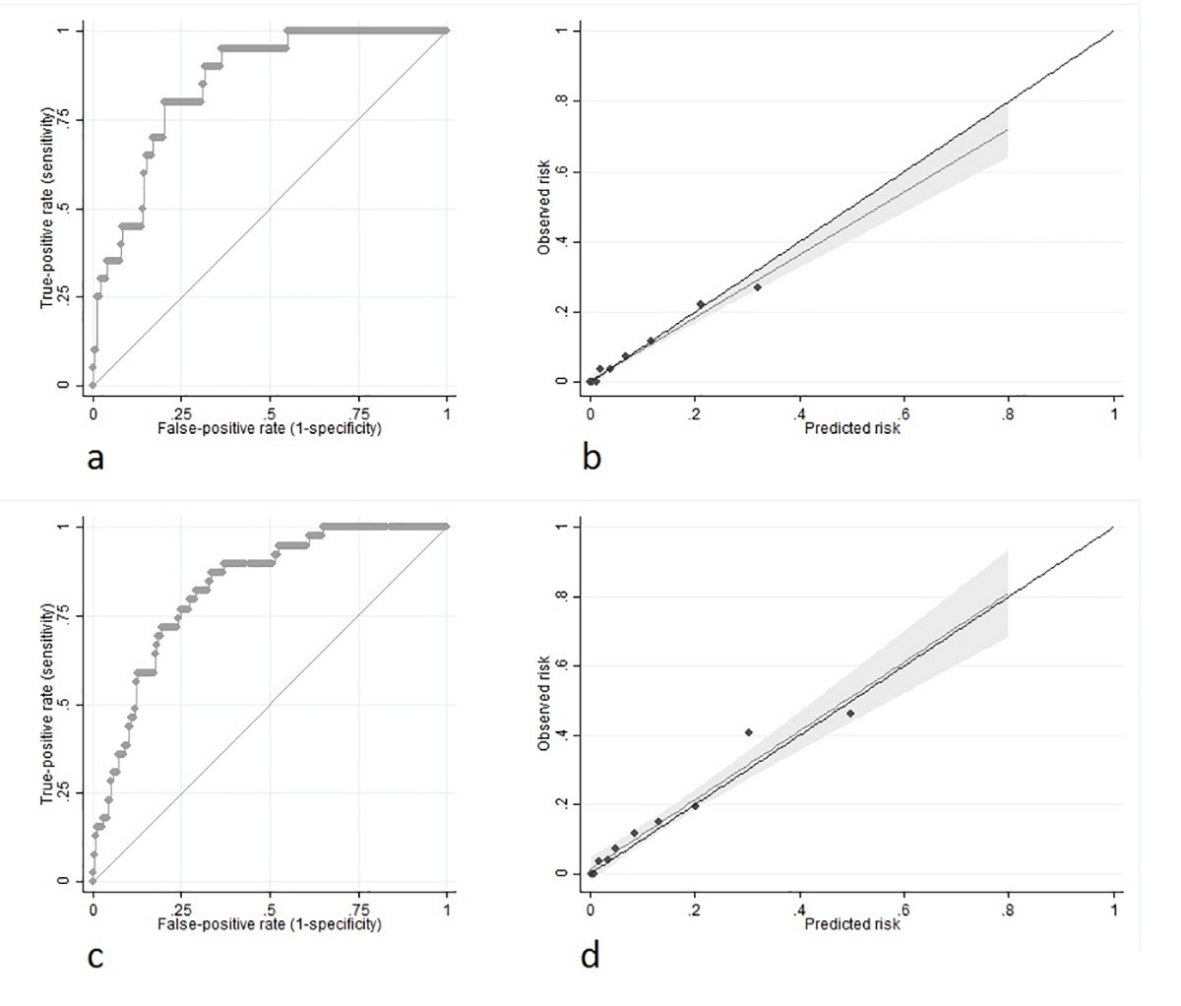
Discrimination and calibration of prediction models in external validation. a) 7-day mortality: Receiver operator characteristic (ROC) curve for discrimination. Area under the curve (AUC) = 0.851 (95%CI 0.781–0.921). b) 7-day mortality: Calibration plot of observed versus predicted risk of mortality (Hosmer-Lemeshow chi-squared = 9.03, p = 0.340; DF = 8). c) 14-day mortality: Receiver operator characteristic (ROC) curve for discrimination. Area under the curve (AUC) = 0.825 (95%CI 0.764–0.887). d) 14-day mortality: Calibration plot of observed versus predicted risk of mortality (Hosmer-Lemeshow chi-squared = 7.63, p = 0.471; DF = 8).

The same prediction models were used to predict each patient's probability of 7-day and 14-day mortality within the external validation cohort. The model had excellent discrimination for 7-day mortality with an AUC of 0.851 (95%CI 0.781–0.921; Fig 4A), as well as an excellent discrimination for 14-day mortality with an AUC of 0.825 (95%CI 0.764–0.887; Fig 4C).

The models showed excellent calibration for predicting both 7-day (Hosmer-Lemeshow chi-squared = 9.03, p = 0.340; DF = 8) and 14-day (Hosmer-Lemeshow chi-squared = 7.63, p = 0.471; DF = 8) mortality, with excellent agreement between observed and predicted risk of mortality across 8 quintiles of risk (Fig 4B and 4D).

## Discussions

The COVID-AID risk tool is a prediction model that accurately estimates the 7- and 14-day risk of death following admission for patients hospitalized with COVID-19 using four simple, well-defined variables that are all available at initial presentation–patient age, mean arterial pressure, serum creatinine and severity of hypoxia. We demonstrated that this prognostic model had consistent test performance in forecasting mortality risk using an independent COVID-19 positive population from another U.S. region in external validation. While other groups around the world have attempted prognostic modeling for COVID-19 disease severity or mortality, a recent systematic review found a lack of generalizability, poor reporting, and severe biases limiting their use [10]. In addition, while some studies have established the severity of respiratory distress by applying a scoring system (Brescia-COVID Respiratory Severity Scale) based on oxygenation status and chest imaging among a critically-ill Italian population affected with COVID-19 [18, 19], there remains great need for a precise prediction score based on variables available at first encounter in order to add clinical meaningfulness and practicality. More recently, a prediction score, COVID-GRAM, was constructed in China to predict development of critical illness among hospitalized patients with COVID-19 [20]. While this 10-item prediction rule was found to have good predictive value (AUC 0.88), there are several limitations that may affect its applicability and generalizability. The study and model’s endpoint, an aggregate outcome of ICU admission and intubation, may be affected by non-clinical factors such as differences in local policy, demand, and resources available. Furthermore, the hospitalized COVID-19 population may also be different in China compared to U.S., as threshold for admission may vary. Certain factors in the prediction rule, such as imaging results or state of consciousness, are also subject to interpretation of the user, thereby adding another layer of subjectivity. These factors may all limit the generalizability of this prediction score, particularly among the U.S. hospitalized COVID-19 population. Therefore, we sought to address this need by developing the COVID-AID 7-day and 14-day mortality risk tool and validated the discrimination and calibration using two independent U.S. populations with COVID-19 disease.

The COVID-AID risk tool is an easy-to-use, bedside clinical decision instrument that may assist healthcare workers in determining resource utilization and hospital triage of patients infected with SARS-CoV2. Additionally, the calculator may also allow patients, family members, and additional caregivers to gain helpful insight on disease severity and prognosis. Moreover, we propose that the COVID-AID risk tool might assist future therapeutic trial design as a validated tool to be used for risk stratification. The strength of this instrument is the simplicity of the variables used in the model, including the generalizability of included admission vitals, age, and serum creatinine, all of which can be readily obtained at all hospitals at the time of hospital or emergency room presentation (See case examples in S3 Table in S1 File). The use of only objective parameters would also help reduce inter-user variability.

Regarding the comprised model variables, each has scientific rationale in the pathophysiology of COVID-19 disease, which strengthens the generalizability of the model (See predicted mortality risks in S1 and S2 Figs). Not surprisingly, the degree of hypoxia at presentation has been well-defined as a significant indicator of severity of illness, particularly in acute respiratory stress conditions, and carries strong justification to be a significant risk factor in the clinical course of severe COVID-19 [18, 19, 21, 22]. In addition, given our focus on short-term mortality as an outcome, we found that lower MAP at presentation was linked with early death and remained a consistent, early adverse predictor among patients in our cohort. We hypothesize that this is due to the fact that these patients were more likely to be suffering from systemic vasodilatory states, such as sepsis [23] or the inflammatory cytokine storm syndrome, which has been associated with severe COVID-19 and ARDS [24–26]. Interestingly, we also found that kidney dysfunction at presentation (defined as serum creatinine ≥2 mg/dL), regardless of chronicity, was the most significant extra-thoracic organ system to impact short-term mortality among hospitalized patients afflicted with COVID-19. This, too, is not surprising, as kidney dysfunction, particularly acute kidney injury, is associated with increased mortality among critically ill patients [27, 28] and is commonly associated with episodes of hypotension [29]. Further supporting our findings, elevated serum creatinine was observed more often in international COVID-19 cohorts among those who died [5, 7, 30], and a recent study on a large cohort of admitted patients with COVID-19 in New York, USA, reported that 22% of total admissions and more than two thirds of admissions leading to death were complicated by acute kidney injury, making it the most common end-organ failure among the admitted patients with COVID-19 [31]. Kidney dysfunction in COVID-19 is hypothesized to be either a consequence of a direct local inflammatory response on the renal epithelial cell during viral inclusion or indirectly as a result from pro-inflammatory and immune-mediated kidney damage [32, 33]. Lastly, patient age, perhaps the most common risk factor for adverse outcomes in acute and chronic illnesses, maintained significance in our prediction model, and thus, justified prior literature quoting age as an important risk factor in COVID-19 adverse outcomes [10, 30, 33–35]. Therefore, these four variables demonstrated that older patients who present with poor oxygenation, hypotension, and kidney dysfunction have a generalizable and plausible increased risk for short-term demise from COVID-19.

Our study relies on the retrospective collection of clinical and outcome data. However, we used a structured data abstraction tool and increased the generalizability of the results by obtaining data from a large cohort of patients admitted at two different New York City hospitals (a tertiary care and a smaller non-teaching hospital). Furthermore, we externally validated our model in a large healthcare system (composed of both an academic tertiary center as well as affiliated community hospitals) in Massachusetts in an effort to ensure consistency in the model’s performance. The COVID-AID score also provides specific advantages over the recently published COVID-GRAM score developed in China, as it requires less input of data (4 variables versus 10), uses only objective parameters easily obtained upon presentation, and predicts the universal outcome of death with comparable performance (AUC 0.825–0.851). Our model requires global validation; however, we attempted to focus on easily reproducible and generalizable variables for this model that entailed only age, initial vital signs (hypoxia and blood pressure), and one laboratory test (serum creatinine) that can be easily obtained in a uniform fashion from a variety of healthcare settings.

In conclusion, the COVID-19 pandemic continues to wage a catastrophic burden on international healthcare and the global economy. As research continues to elucidate effective therapies for SARS-CoV2, healthcare workers and patients alike need assistance in understanding what clinical parameters on admission might predict increased severity of disease and short-term mortality. We have developed an easy-to-use clinical prognostic score that accurately predicts risk of mortality with excellent calibration and consistency in test performance using an external validation cohort. The COVID-AID risk tool calculator is also available online at www.covidaidscore.com. While there is need for international validation, this novel mortality prediction model may help providers understand the expected risk of death for patients presenting to the hospital. We propose that the COVID-AID risk tool can enhance our knowledge of how to successfully manage these patients, lead to more effective healthcare resource utilization, and provide patients and their loved ones with improved understanding of disease severity and prognosis. Additionally, the COVID-AID risk tool also delivers an accurate risk stratification estimate for researchers to properly design future trials in hopes of discovering effective therapies against this virus.

## Supporting information

S1 Fig 7-day risk of mortality in patients admitted with COVID-19, based on age (18 to 80 years), mean arterial pressure (MAP, 40 to 100 mmHg), severe hypoxia, and kidney dysfunction. a) No severe hypoxia or kidney dysfunction. b) No severe hypoxia, but kidney dysfunction present. c) Severe hypoxia present, but no kidney dysfunction. d) Both severe hypoxia and kidney dysfunction present.
(JPG)

S2 Fig 14-day risk of mortality in patients admitted with COVID-19, based on age (18 to 80 years), mean arterial pressure (MAP, 40 to 100 mmHg), severe hypoxia, and kidney dysfunction. a) No severe hypoxia or kidney dysfunction. b) No severe hypoxia, but kidney dysfunction present. c) Severe hypoxia present, but no kidney dysfunction. d) Both severe hypoxia and kidney dysfunction present.
(JPG)

S1 File (DOCX)

S2 File (XLSX)

## Abbreviations

AID: Admission to Death
COVID-19: 2019 novel coronavirus disease
SARS-CoV-2: severe acute respiratory syndrome coronavirus 2
RNA: ribonucleic acid
ARDS: Acute respiratory distress syndrome
AST: aspartate aminotransferase
ALT: alanine aminotransferase
NIPPV: non-invasive positive pressure ventilation
ICU: intensive care unit
BMI: body mass index
SD: standard deviation
CI: confidence interval
CHAID: Chi-square automatic interaction detection
ROC: receiver operating characteristic
AUC: Area under the curve
OR: odds ratio
IQR: Interquartile range
