# Supplementary figures and images for: Development and external validation of a prediction risk model for short-term mortality among hospitalized U.S. COVID-19 patients: A proposal for the COVID-AID risk tool
Source: PLoS One. 2020 Sep 30;15(9):e0239536. doi: 10.1371/journal.pone.0239536 (PMC7526907; doi:10.1371/journal.pone.0239536)

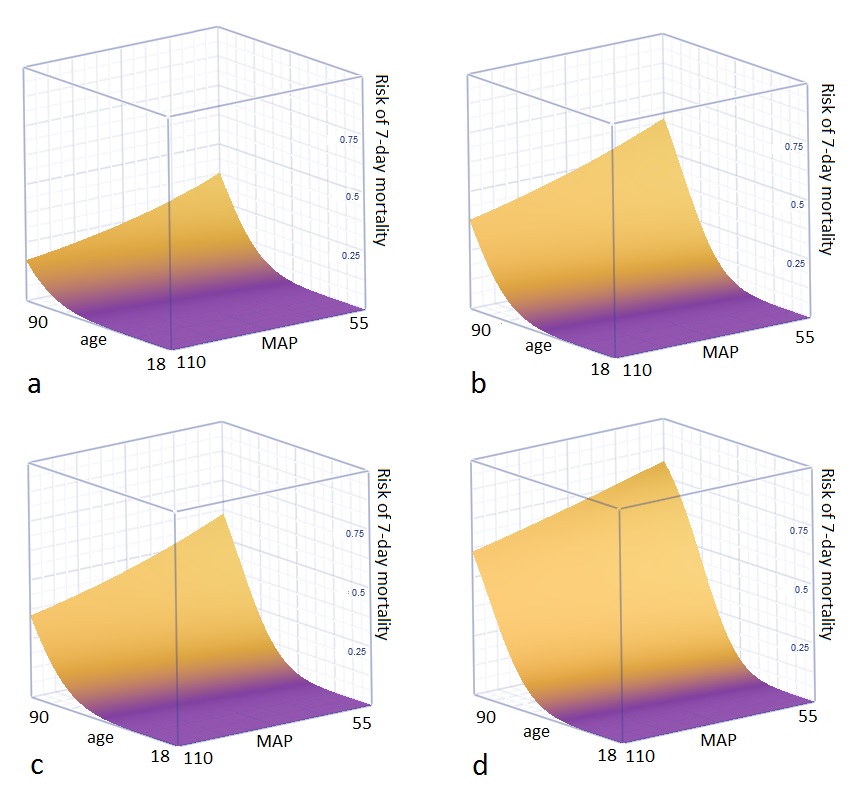

Supplement: S1 Fig — a) No severe hypoxia or kidney dysfunction. b) No severe hypoxia, but kidney dysfunction present. c) Severe hypoxia present, but no kidney dysfunction. d) Both severe hypoxia and kidney dysfunction present. (JPG) [file pone.0239536.s001.jpg]

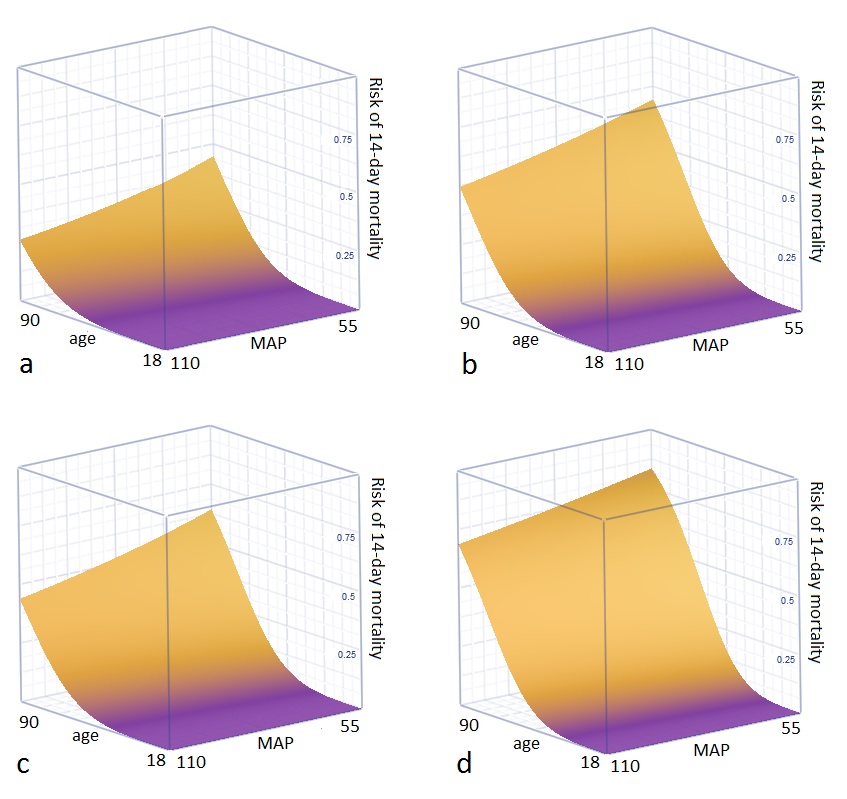

Supplement: S2 Fig — a) No severe hypoxia or kidney dysfunction. b) No severe hypoxia, but kidney dysfunction present. c) Severe hypoxia present, but no kidney dysfunction. d) Both severe hypoxia and kidney dysfunction present. (JPG) [file pone.0239536.s002.jpg]
