# Supplementary material for: Development and external validation of a prediction risk model for short-term mortality among hospitalized U.S. COVID-19 patients: A proposal for the COVID-AID risk tool
Source: PLoS One. 2020 Sep 30;15(9):e0239536. doi: 10.1371/journal.pone.0239536 (PMC7526907; doi:10.1371/journal.pone.0239536)
Supplement: S1 File — (DOCX) [file pone.0239536.s003.docx]

**Supplementary table 1.** TRIPOD checklist for transparent reporting on a multivariable prognostic model.

| Section/topic | Item | Checklist item | Page |
| --- | --- | --- | --- |
| Title and abstract | | | |
| Title | 1 | Identify the study as developing and/or validating a multivariable prediction model, the target population, and the outcome to be predicted. | 1 |
| Abstract | 2 | Provide a summary of objectives, study design, setting, participants, sample size, predictors, outcome, statistical analysis, results, and conclusions. | 2 |
| Introduction | | | |
| Background and objective | 3a | Explain the medical context (including whether diagnostic or prognostic) and rationale for developing or validating the multivariable prediction model, including references to existing models. | 4 |
|  | 3b | Specify the objectives, including whether the study describes the development or validation of the model or both. | 5 |
| Methods | | | |
| Source of data | 4a | Describe the study design or source of data (e.g., randomized trial, cohort, or registry data), separately for the development and validation data sets, if applicable. | 5,6 |
|  | 4b | Specify the key study dates, including start of accrual; end of accrual; and, if applicable, end of follow-up. | 5,8 |
| Participants | 5a | Specify key elements of the study setting (e.g., primary care, secondary care, general population) including number and location of centres. | 5,8 |
|  | 5b | Describe eligibility criteria for participants. | 5,6 |
|  | 5c | Give details of treatments received, if relevant. | NA |
| Outcome | 6a | Clearly define the outcome that is predicted by the prediction model, including how and when assessed. | 7 |
|  | 6b | Report any actions to blind assessment of the outcome to be predicted. | NA |
| Predictors | 7a | Clearly define all predictors used in developing or validating the multivariable prediction model, including how and when they were measured. | 5-7 |
|  | 7b | Report any actions to blind assessment of predictors for the outcome and other predictors. | NA |
| Sample size | 8 | Explain how the study size was arrived at. | NA |
| Missing data | 9 | Describe how missing data were handled (e.g., complete-case analysis, single imputation, multiple imputation) with details of any imputation method. | 9, Supplementary table 3 |
| Statistical analysis | 10a | Describe how predictors were handled in the analyses. | 7-9 |
|  | 10b | Specify type of model, all model-building procedures (including any predictor selection), and method for internal validation. | 7-14 |
|  | 10d | Specify all measures used to assess model performance and, if relevant, to compare multiple models. | 11-13 |
| Risk groups | 11 | Provide details on how risk groups were created, if done. | 11-13 |
| Results | | | |
| Participants | 13a | Describe the flow of participants through the study, including the number of participants with and without the outcome and, if applicable, a summary of the follow-up time. A diagram may be helpful. | Figure 1 |
|  | 13b | Describe the characteristics of the participants (basic demographics, clinical features, available predictors), including the number of participants with missing data for predictors and outcome. | 9, 10, Table 1, Figure 1 |
| Model development | 14a | Specify the number of participants and outcome events in each analysis. | 9,  Figure 1, Table 1 |
|  | 14b | If done, report the unadjusted association between each candidate predictor and outcome. | Table 1 |
| Model specification | 15a | Present the full prediction model to allow predictions for individuals (i.e., all regression coefficients, and model intercept or baseline survival at a given time point). | 12, Table 2 |
|  | 15b | Explain how to the use the prediction model. | 12,15 |
| Model performance | 16 | Report performance measures (with CIs) for the prediction model. | 12-14, Figure 3a-d, Figure 4a-d |
| Discussion | | | |
| Limitations | 18 | Discuss any limitations of the study (such as nonrepresentative sample, few events per predictor, missing data). | 17, Supplementary table 3 |
| Interpretation | 19b | Give an overall interpretation of the results, considering objectives, limitations, and results from similar studies, and other relevant evidence. | 14-18 |
| Implications | 20 | Discuss the potential clinical use of the model and implications for future research. | 14-18, Supplementary Table 2 |
| Other information | | | |
| Supplementary information | 21 | Provide information about the availability of supplementary resources, such as study protocol, Web calculator, and data sets. | 17 |
| Funding | 22 | Give the source of funding and the role of the funders for the present study. | 1 |

**Supplementary table 2.** Details of non-missing data (available for complete case analysis) on demographics, medical history, laboratory, and clinical findings of patients with COVID-19 upon admission.

| Variable | Total, n=664 | Death within 14 days of admission | |
| --- | --- | --- | --- |
|  |  | No, n=571 | Yes, n=93 |
|  | Non-missing, n(%) | | |
| Age, years | 664(100) | 571(100) | 93(100) |
| Gender | 664(100) | 571(100) | 93(100) |
| Race/ethnicity | 492(74) | 424(74) | 68(73) |
| BMI | 479(72) | 414(73) | 65(70) |
| Pre-existing comorbidities |  |  |  |
| Hypertension | 664(100) | 571(100) | 93(100) |
| Diabetes | 664(100) | 571(100) | 93(100) |
| Chronic kidney disease | 664(100) | 571(100) | 93(100) |
| Cardio-vascular disease | 664(100) | 571(100) | 93(100) |
| COPD/Asthma | 664(100) | 571(100) | 93(100) |
| Obstructive sleep apnea | 664(100) | 571(100) | 93(100) |
| VTE | 664(100) | 571(100) | 93(100) |
| Cancer | 664(100) | 571(100) | 93(100) |
| IBD | 664(100) | 571(100) | 93(100) |
| Chronic liver disease | 664(100) | 571(100) | 93(100) |
| Solid organ transplantation | 664(100) | 571(100) | 93(100) |
| Vital signs |  |  |  |
| fever | 663(100) | 570(100) | 93(100) |
| Respiratory rate | 658(99) | 565(99) | 93(100) |
| Heart rate | 662(100) | 569(100) | 93(100) |
| Mean arterial pressure | 664(100) | 571(100) | 93(100) |
| Hypoxia on presentation | 664(100) | 571(100) | 93(100) |
| Kidney dysfunction | 664(100) | 571(100) | 93(100) |
| Laboratory findings |  |  |  |
| Creatinine | 664(100) | 571(100) | 93(100) |
| White blood cell count | 658(99) | 565(99) | 93(100) |
| Absolute lymphocyte count | 646(97) | 554(97) | 92(99) |
| Absolute neutrophil count | 645(97) | 553(97) | 92(99) |
| Platelet count | 659(99) | 566(99) | 93(100) |
| Procalcitonin | 567(85) | 485(85) | 82(88) |
| D-dimer | 365(55) | 314(55) | 51(55) |
| C reactive protein | 484(73) | 414(73) | 70(75) |
| Lactate dehydrogenase | 507(76) | 437(77) | 70(75) |
| Lactate | 396(60) | 331(58) | 65(70) |
| Ferritin | 431(65) | 369(65) | 62(67) |
| Troponin I | 511(77) | 429(75) | 82(88) |
| Albumin | 641(97) | 549(96) | 92(99) |
| Total bilirubin | 642(97) | 550(96) | 92(99) |
| ALT | 642(97) | 550(96) | 92(99) |
| AST | 638(96) | 549(96) | 89(96) |
| Alkaline phosphatase | 641(97) | 550(96) | 91(98) |

VTE: venous thromboembolism; IBD: inflammatory bowel disease; NSAID: non-steroidal anti-inflammatory drug; AST: aspartate aminotransferase; ALT: alanine aminotransferase; INR: international normalized ratio; aPTT: activated partial thromboplastin time.

**Supplementary table 3.** Case examples of predicted 7- and 14-day mortalities based on patients' characteristics at time of admission.

| Patient characteristics on admission | | | | Predicted mortality | |
| --- | --- | --- | --- | --- | --- |
| Age | MAP* | Severe hypoxia | Kidney dysfunction^ | 7-day, % | 14-day, % |
| 40 | 65 | Yes | No | 0.2 | 0.8 |
| 55 | 65 | No | Yes | 2.7 | 7.4 |
| 55 | 55 | Yes | Yes | 8.6 | 17.7 |
| 80 | 55 | Yes | Yes | 64.2 | 70.3 |

*MAP: mean arterial pressure. ^ Kidney dysfunction defined as serum creatinine≥ 2 mg/dL.
